# Restorative and Periodontal Outcomes of Deep Margin Elevation in Posterior Teeth: A Systematic Review

**DOI:** 10.7759/cureus.97278

**Published:** 2025-11-19

**Authors:** Prerna Priya, Ankita Singh, Ashi Chug

**Affiliations:** 1 Conservative Dentistry and Endodontics, All India Institute of Medical Sciences, Rishikesh, IND; 2 Dentistry, Employees' State Insurance Corporation (ESIC) Medical College and Hospital, Faridabad, IND; 3 Conservative Dentistry and Endodontics, ITS Dental College Hospital and Research Centre, Greater Noida, IND; 4 Dentistry, All India Institute of Medical Sciences, Rishikesh, IND

**Keywords:** cervical margin relocation, deep margin elevation, periodontal health, restorative dentistry, subgingival margin

## Abstract

Deep margin elevation (DME) is a minimally invasive restorative technique that relocates subgingival margins coronally to facilitate adhesive bonding and isolation. It is proposed as a conservative alternative to surgical crown lengthening for teeth with deep cervical margins. The present systematic review evaluated the restorative success and periodontal response of DME, with emphasis on long-term restoration survival, marginal adaptation, and periodontal health. Electronic searches of PubMed, Scopus, Web of Science, Embase, and CENTRAL were conducted from database inception to August 2025. Randomized controlled trials (RCTs), non-randomized clinical trials, controlled clinical trials, and retrospective studies reporting restorative or periodontal outcomes after DME were included. Eight clinical studies (n = 678) were included: two RCTs, three non-randomized clinical trials, two retrospective cohorts, and one controlled clinical trial. Follow-up ranged from three months to 12 years. Restoration survival was consistently high, with long-term success rates of 94%-100% and slightly higher survival for ceramic than for composite restorations. Pocket depth remained within healthy limits (1.9 to 2.8 mm) across all studies. Early increases in bleeding on probing (up to 53% at 12 months) and plaque index were observed, but stabilized or decreased over time with adequate hygiene. Clinical attachment loss remained stable or showed slight improvement in long-term follow-up. DME yields excellent long-term restorative outcomes and maintains periodontal health when performed with meticulous adhesive technique and appropriate maintenance. Findings of the present systematic review support its use as a conservative alternative to surgical crown lengthening.

## Introduction and background

When dental caries, fractures, or previous restorations extend below the gingival margin, the dentist faces the complex challenge of restoring an area that lies partly within the biologically active gingival sulcus. Subgingival margins are continuously bathed in gingival crevicular fluid, which contains inflammatory mediators and microbial components that can interfere with adhesive bonding, compromise polymerization of resin-based materials, and weaken the marginal seal of restorations. Inadequate sealing at these margins allows bacterial biofilm accumulation and fluid ingress, leading to microleakage, marginal discoloration, and potential loss of periodontal attachment [1]. The interplay between restorative materials and the subgingival environment is therefore critical to both restorative longevity and periodontal health.

Conventional management approaches, such as surgical crown lengthening or orthodontic extrusion, are designed to expose sound tooth structure and re-establish a healthy biologic width - the combined dimension of the junctional epithelium and connective tissue attachment that separates the gingival margin from the alveolar crest [2]. While these procedures can improve access and visibility for restorative work, they are invasive and often lead to collateral changes in bone and soft-tissue architecture, potentially compromising esthetics, crown-root ratio, and periodontal stability. Surgical manipulation may also alter the local microbiome and influence gingival healing patterns, especially when oral hygiene is suboptimal [3,4].

In response to these limitations, deep margin elevation (DME), also referred to as cervical margin relocation, has emerged as a minimally invasive restorative alternative [5]. This technique involves relocating a deep subgingival margin to a more accessible supragingival level using adhesive restorative materials such as flowable composites, resin-modified glass ionomer (RMGI), or glass hybrids. By creating a coronal relocation of the margin, DME facilitates isolation, adhesive bonding, and impression making while preserving tooth structure and maintaining the original periodontal attachment apparatus [4,5]. The approach is particularly valuable in posterior teeth where surgical access is limited and esthetic concerns are secondary.

However, the periodontal and biological implications of DME remain an area of debate. The close proximity of restorative materials to the gingival tissues raises questions about plaque accumulation, soft-tissue inflammation, and long-term periodontal stability. Clinical reports have shown mixed findings - some demonstrating excellent restoration survival and stable periodontal health, while others noted temporary increases in bleeding or plaque indices during early follow-up periods [6-8]. These inconsistencies may stem from differences in margin position, restorative material selection, finishing protocols, and follow-up duration.

The present systematic review aims to synthesize current clinical evidence on both restorative and periodontal outcomes associated with DME. The goal of the review is to clarify whether DME provides a biologically safe and durable alternative to more invasive crown-lengthening procedures, inform material and technique selection, and identify gaps that warrant future research in adhesive restorative dentistry.

## Review

Methodology

Protocol and Registration

The present systematic review was planned and conducted in accordance with the Preferred Reporting Items for Systematic Reviews and Meta-Analyses (PRISMA 2020) statement [9]. A protocol detailing the review question, eligibility criteria, and methods of data collection and analysis was prepared before initiating the review and registered with the International Prospective Register of Systematic Reviews (PROSPERO) database (Reference ID: CRD420251147537)

Information Sources and Search Strategy

A comprehensive electronic search was undertaken to identify relevant literature from database inception until August 2025. The following databases were searched: PubMed/MEDLINE, Scopus, Web of Science, Embase, and the Cochrane Central Register of Controlled Trials (CENTRAL). To capture grey literature and unpublished data, Google Scholar and OpenGrey were searched, and the reference lists of all included articles and relevant reviews were screened for additional studies. The electronic search strategy combined free-text terms related to deep margin elevation and cervical margin relocation. A representative PubMed search was executed on August 15 which included (“deep margin elevation”[Title/Abstract] OR “cervical margin relocation”[Title/Abstract] OR “margin relocation”[Title/Abstract]) AND (“composite”[Title/Abstract] OR “adhesive”[Title/Abstract] OR “resin modified glass ionomer”[Title/Abstract] OR “RMGI”[Title/Abstract] OR “glass hybrid”[Title/Abstract]) AND (“periodontal”[Title/Abstract] OR “gingival”[Title/Abstract] OR “plaque”[Title/Abstract] OR “probing”[Title/Abstract] OR “survival”[Title/Abstract] OR “adaptation”[Title/Abstract]). The strategy was adapted for use in other databases to account for differences in indexing and syntax (Appendix 1).

Eligibility Criteria

Studies were considered eligible if they were randomized controlled trials (RCTs), non-randomized clinical trials, prospective or retrospective observational studies, or controlled clinical trials that evaluated DME in posterior teeth and reported at least one periodontal or restorative outcome of interest. Case series, case reports, narrative reviews, editorials, conference abstracts lacking full data, and purely in vitro or animal studies were excluded. Adult patients aged 18 years or older undergoing adhesive restorative treatment with cervical margin relocation were eligible, regardless of tooth vitality. The intervention of interest was DME performed using adhesive restorative materials, including but not limited to composite resins, glass hybrids, resin-modified glass ionomer, or other flowable or packable composite systems. Both direct and indirect DME techniques were included. Comparator groups, when present, included conventional preparations without DME or baseline measurements in single-arm studies. There was no minimum follow-up requirement for inclusion, provided that at least one post-operative evaluation was reported.

Study Selection

A standardized screening form was pilot-tested on 25 randomly selected titles/abstracts to ensure consistency (Appendix 2). Two reviewers (PP and AS) screened all records independently, documenting reasons for exclusion at full-text stage using predefined categories (irrelevant design, non-posterior teeth, in vitro study, insufficient outcome data). The screening form and data-extraction sheet are provided in Appendix 2. All identified records were imported into the Elicit systematic review tool for duplicate removal. Two reviewers independently screened the titles and abstracts of all retrieved articles to identify potentially eligible studies. Full texts of potentially relevant articles were obtained and assessed in detail against the inclusion criteria. Any disagreements between reviewers were resolved through discussion and, when necessary, adjudication by a third reviewer. 

Data Extraction

Data were extracted independently and in duplicate by two reviewers using a standardized data extraction form that had been pilot tested before use. The extracted information included study identifiers (author, year of publication, and country), design, sample size, participant characteristics such as mean age, standard deviation, and gender distribution, intervention details including restorative materials, bonding systems, technique of DME (direct or indirect), and location of margin elevation, and comparator details where available. Clinical outcome parameters, including restorative outcomes such as marginal adaptation, radiographic adaptation, and survival or success rates of restorations, and periodontal measures, including bleeding on probing (BoP), gingival index (GI), plaque index (PI), probing depth (PD), clinical attachment level (CAL), full-mouth plaque and bleeding scores, and papillary or gingival bleeding indices, were recorded. Baseline and follow-up values, ranges, standard deviations, and statistical comparisons were extracted when available. The reviewers cross-checked the extracted data to ensure accuracy and completeness.

Data Synthesis and Statistical Analysis

Due to substantial heterogeneity across study designs, restorative materials, outcome measures, and follow-up durations, a narrative synthesis was adopted as the primary method of data integration. A meta-analysis was planned only if at least three studies reported comparable quantitative outcomes with consistent measurement methods and variance estimates. Heterogeneity was assessed both conceptually (by evaluating study design, intervention characteristics, and outcome definitions) and numerically (by reviewing reported mean values, standard deviations, and ranges). However, inconsistencies in reported metrics, absence of standard deviations or confidence intervals in several studies, and variable follow-up periods (three months - 12 years) precluded reliable data pooling.

Continuous outcomes (e.g., probing depth, gingival index, plaque index, clinical attachment level) were summarized as means ± standard deviations when reported. Categorical data (e.g., survival rate, bleeding on probing) were summarized as proportions or event rates. P-values and 95% confidence intervals have been reported wherever available. Where these data were missing, results were described qualitatively with emphasis on direction and magnitude of change rather than statistical significance. Because the dataset did not meet the criteria for statistical homogeneity or sufficient variance reporting, a formal quantitative meta-analysis was not statistically appropriate. Therefore, no forest plots or pooled estimates were generated.

Assessment of Risk of Bias

The methodological quality of all included studies was appraised independently by two reviewers using tools appropriate to each study design. Randomized controlled trials were evaluated using the Cochrane Risk of Bias 2 (RoB-2) tool, which assesses potential bias arising from the randomization process, deviations from intended interventions, missing outcome data, outcome measurement, and selective reporting of results [10]. Non-randomized clinical trials were assessed with the Risk of Bias in Non-randomized Studies of Interventions (ROBINS-I) tool, focusing on confounding, participant selection, classification of interventions, deviations from intended interventions, missing data, outcome measurement, and reporting bias [11]. Retrospective observational studies were appraised using the Newcastle-Ottawa Scale (NOS), which evaluates cohort selection, comparability of study groups, and adequacy of outcome assessment [12]. Each study was reviewed in duplicate, and any disagreements were resolved through discussion and consensus. Domain-level judgments were recorded as low risk, some concerns, moderate risk, or serious risk for RoB-2 and ROBINS-I, and as NOS scores. 

Results

Study Characteristics

**Figure 1:**
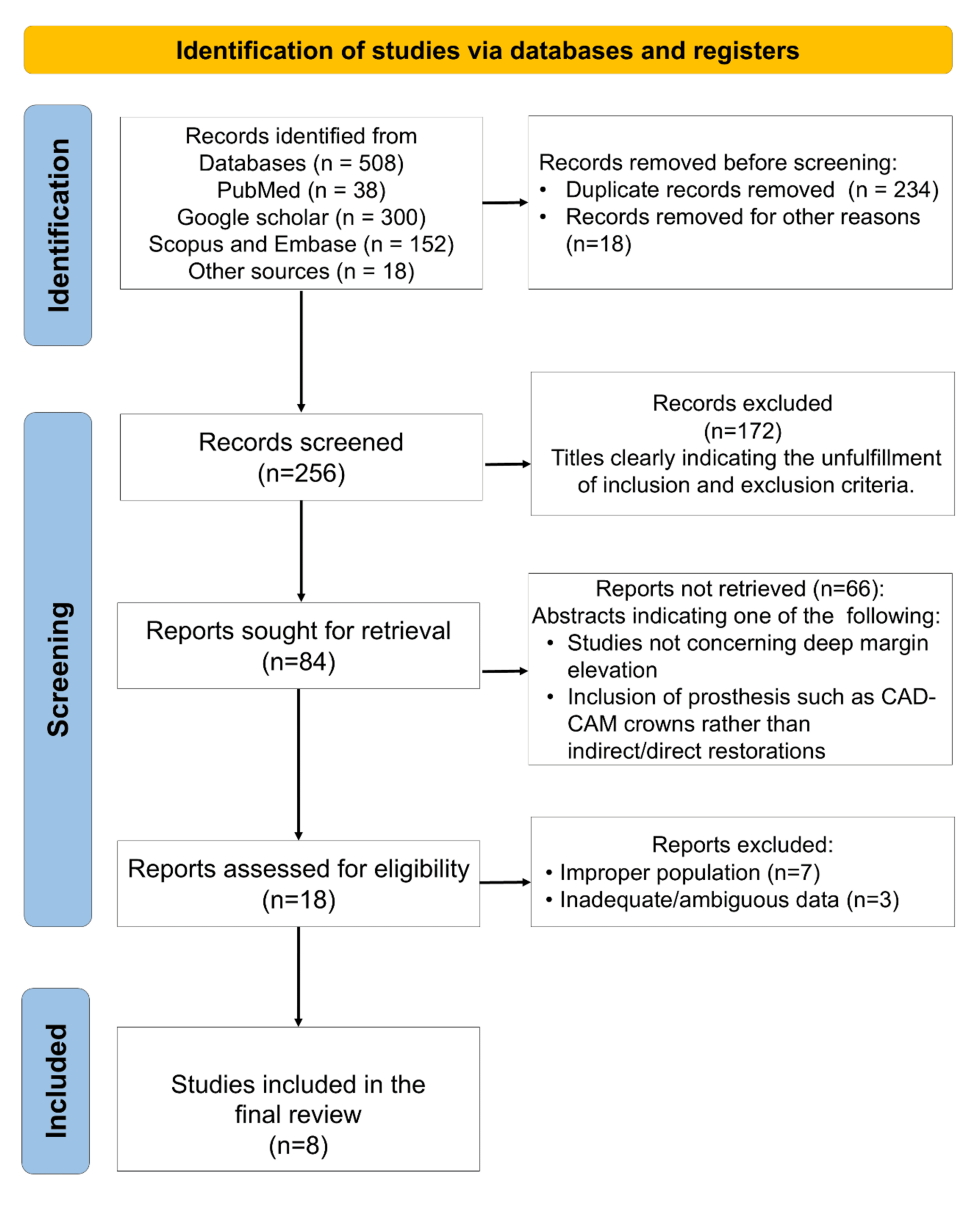
Preferred Reporting Items for Systematic Reviews and Meta-Analyses (PRISMA) Flow Diagram depicting the article selection process

From an initial pool of 508 screened records, eight clinical studies met the inclusion criteria for this systematic review (Figure 1). The numeric summary of screening and inclusion is provided in Appendix 3. These comprised three RCTs (Ferrari 2017; Ismail 2023; Farouk 2023), three non-randomized clinical trials (Ghezze 2019; Bertoldi 2019; Bresser 2019), and two retrospective observational studies (Muscholl 2022; El-Ma’aita 2024) [13-20]. Together, these studies represented a total of n = 678 participants, with individual sample sizes ranging from 15 to 197. The data extracted from the included studies are collectively summarized in Table 1.

**Table 1:** Data extracted from the studies included in the present systematic review BoP – Bleeding on Probing; GI – Gingival Index; PI – Plaque Index; PD – Probing Depth; CAL – Clinical Attachment Level; CBL – Crestal Bone Level; FMPS – Full-Mouth Plaque Score; FMBS – Full-Mouth Bleeding Score; GBI – Gingival Bleeding Index; NS – Not Significant; DME – Deep Margin Elevation; CL – Crown Lengthening; OR – Odds Ratio; CI – Confidence Interval; RMGI – Resin-Modified Glass Ionomer Quantitative pooling (meta-analysis) was not performed because of inconsistent outcome definitions, incomplete variance data, and heterogeneous follow-up durations among studies.

Author (Year)	Country	Study Design	N	Restorative Material	Technique	Margin Location	Restoration Type	Clinical Parameters	Reported Significance	Follow-up	Key Quantitative Results	Statistical Significance (p, 95 % CI)	Conclusions (Margins / Periodontium)
M. Ferrari et al. (2017)	Italy	RCT (split-mouth)	35	G-Premio Bond + universal flow resin composite	Direct DME	Supra-gingival	Partial	BoP, GI, PI	BoP ↑ in CMR vs control (p = 0.010)	BL, 12 mo	Baseline BoP & GI = 0 %; 12 mo BoP = 53 % (CMR) vs 31.5 % (Shoulder); bone crest → margin = 2 mm in 13/19 (CMR) vs 6/11 (Shoulder)	BoP higher in CMR (p = 0.010); PPD mesial/distal baseline (p = 0.001)	Higher BoP at 12 mo around elevated margins
Bertoldi et al. (2019)	Italy	Non-randomized trial	29	Composite (Esthet-X HD)	Direct DME	Sub-gingival	Partial (pre-endo)	FMPS, FMBS, PD	PD reduction significant	BL, 3 mo	FMPS 13.01 → 11.48 %; FMBS 9.27 → 7.48 %; PD A 1.95 → 1.66 mm; B 2.57 → 2.21 mm	FMPS, FMBS, PD ↓ (p < 0.05–0.01); CM-AMR vs CM-APP (p < 0.05)	Subgingival DME produced stable attachment and reduced bleeding
Painz et al. (2015)	—	Controlled trial	—	—	—	—	—	PI, GI, PD, BoP	—	BL, 12 mo	BoP: Chamfer 36.5 %, Feather 52.2 %; GI ↑ 31–33 %; No recession: Chamfer 88.5 %, Feather 96.7 %	BoP ↑ Feather (p = 0.028); less recession (p = 0.03); PI/GI/PD ↓ (p < 0.05)	Feather-edge margins showed more bleeding but less recession
Ghezze et al. (2019)	Italy	Non-randomized trial	15	Nanohybrid composite	Direct DME	Supra-gingival	Indirect composite	PD, BoP	NS among groups	5.7 yrs	Overall PD 2.5 → 2.3 mm; BoP ↓ 100 % → 40 % (1 yr)	No significant findings reported	Stable PD and improved bleeding scores long-term
R. A. Bresser et al. (2019)	Netherlands	Non-randomized trial	197	Hybrid nanofilled composite	Direct DME	Supra-gingival	Partial indirect	Survival, periodontal	—	12 yrs	Survival 95.9 % (SE 2.9 %); χ² = 9.02–42.03 for defects	Material (p = 0.000–0.04); fracture χ² = 42.03 (p = 0.000); wear χ² = 6.62 (p = 0.04)	High survival and periodontal stability > 10 yrs
Muscholl et al. (2022)	Germany	Retrospective	63	Optibond FL + Tetric Evo Ceram	Direct DME	Sub-gingival	Direct composite	PD, CAL, BoP, GBI	—	3 yrs	CAL +0.421 mm vs control (p = 0.027); GBI ↑ no brush use (p = 0.010, OR 6.29 [1.57–11.01]); BoP linked to CAL (p < 0.001)	CAL ↑ (p = 0.027); GBI ↑ (p = 0.010); OR 6.29 (95 % CI 1.57–11.01)	Slight CAL increase without added inflammation
Hoda S. Ismail et al. (2023)	Egypt	RCT	95 pts / 120 restos	RMGI, Glass Hybrid, Bulk-fill Resin, Activa	Direct DME	Sub-gingival	Full	PI, GIx, PBI, PD	Radiographic adaptation NS	3 yrs	PI RMGI 21/24 → 2/24; GH 20/24 → 1/24; PD ≈ 2.2 → 2.6 mm; Activa radiographic score ↓ (p < 0.05)	Radiographic (p < 0.05); PD ↑ first year (p < 0.05); GIx/PBI NS	All materials maintained healthy periodontium
Ahmed T. Farouk et al. (2023)	Egypt	RCT	20	Indirect restorations	Direct DME	Sub-→ Supra gingival	Indirect	CAL, PD, CBL	DME < CL (p < 0.05)	12 mo	CAL 3–12 mo DME < CL (p < 0.05); PD 9–12 mo DME < CL (p < 0.05); CBL ↓ (p < 0.05); BoP NS	CAL, PD, CBL ↓ (p < 0.05); BoP NS	Better periodontal response vs CL
A. El-Ma’aita et al. (2024)	Jordan	Retrospective	28	Resin composite (Filtek Z250)	Direct DME	Sub-→ Supra gingival	Partial / Full	PD, BoP, bone level	All NS	25.4 mo	Success 96.6 %; PD 2.8 (DME) vs 2.6 (non-DME) p = 0.34; Bone distance 1–3 mm (mean 1.92 mm)	No significant findings (p > 0.05)	No PD difference or bone loss

Where reported, participants' ages ranged from 24 to 70 years, although several studies did not provide complete demographic information. Gender distribution was inconsistently described; only three studies reported male-to-female ratios [13,15,16]. Most interventions evaluated direct DME performed with adhesive composite or nanohybrid composite materials, while two studies included indirect restorations in which the cervical margin was relocated before final prosthesis placement [15,19]. The location of the elevated margin varied: four studies involved margins relocated to a supra-gingival position, whereas five studies evaluated margins placed at a sub-gingival or sub- to supra-gingival level. Follow-up periods were highly variable, ranging from three months to 12 years, allowing assessment of both early periodontal responses and long-term restorative performance.

Endodontic and Restorative Outcomes

The primary clinical outcome across studies was the success and survival of teeth restored after DME, including those with endodontic treatment or indirect coverage. Results consistently demonstrated good medium- to long-term survival rates irrespective of margin location or restorative material. The largest non-randomized clinical trial (Bresser 2019; n=197) reported a cumulative survival of 95.9% at 12 years, with ceramic restorations performing slightly better than composite (96.5% vs 94.3%) [15]. Presence of a proximal contact improved survival to 97.1% compared with 85.7% when no contact was present. Ghezze (2019) observed 100% survival of indirect composite restorations over a mean of 5.7 years, with stable periodontal parameters [14]. Ismail (2023) recorded no clinical failures over three years across four restorative materials (resin-modified glass ionomer, glass hybrid, bulk-fill resin, and Activa) [18]. El-Ma’aita (2024) reported a 96.6% success rate at a mean of 25.4 months, with no secondary caries, discoloration, or periodontal pocketing and a safe DME-bone distance of 1-3 mm (mean 1.92 mm) [20]. In the RCT by Farouk (2023), which compared DME with surgical crown-lengthening (CL) for teeth requiring indirect full-coverage restorations, secondary caries were absent in both groups over 12 months, while DME demonstrated significantly lower PD and CAL from three to 12 months [19]. Collectively, these findings support DME as a reliable alternative to surgical crown lengthening for preserving tooth structure and ensuring endodontic and restorative success.

Marginal and Radiographic Adaptation

Detailed marginal adaptation outcomes were primarily available from Ismail (2023). Marginal integrity remained stable across all restorative materials, with bulk-fill composites maintaining 20/25 acceptable margins and Activa maintaining 19/23 after three years [18]. Radiographic adaptation was similarly acceptable, with Activa receiving a standardized score of 3, indicating satisfactory adaptation. Other studies confirmed clinically acceptable emergence profiles but did not provide quantitative radiographic data.

Bleeding on probing: BoP outcomes were reported in six studies. Baseline BoP was 0% in all RCTs. Ferrari (2017) observed an increase to 53% in the cervical margin relocation (CMR) group compared with 31.5% in controls at 12 months [13]. In contrast, Ghezze (2019) reported a marked reduction from 100% at baseline to 40% after one year, which remained stable over a mean follow-up of 5.7 years [14]. Bertoldi (2019) noted BoP reductions from 9.27% ± 2.1% to 7.48% ± 2.24% at three months [15]. Muscholl (2022) found no significant increase in BoP at three years but identified a strong correlation between gingival bleeding and lack of interdental brush use (OR = 6.29, p = 0.010) [17]. Farouk (2023) reported no significant BoP differences between DME and crown-lengthening groups up to 12 months [19]. Overall, transient early increases in BoP were mild and reversible with good plaque control.

Gingival index: Gingival inflammation was assessed in five studies. Ferrari (2017) reported GI increases to 31.5% in the CMR group and 18.5% in controls at 12 months [13]. Ismail (2023) observed non-significant fluctuations across materials over three years: for instance, in the Activa group, positive GIx sites declined from 11/23 at baseline to 6/23 at three years, while RMGI decreased from 13/24 to 7/24 [18]. El-Ma’aita (2024) reported no significant differences in gingival inflammation between DME and non-DME sites [20]. These results indicate that DME margins do not induce sustained gingival inflammation when restorative surfaces are well finished and patients maintain hygiene.

Plaque index: Plaque accumulation was consistently low across the studies. In Ismail (2023), all four restorative materials showed a marked decrease in positive PI scores during follow-up [18]. Ferrari (2017) recorded a mild increase to 20% in the CMR group versus 8.5% in controls at 12 months [13]. El-Ma’aita (2024) found mean plaque levels similar between DME and non-DME sites (p = 0.34) [20]. Overall, long-term PI outcomes showed stabilization or decline, confirming that properly contoured DME margins do not promote plaque retention.

Probing depth: PD values were reported in almost all included studies. Ghezze (2019) documented sustained improvement, with mean PD decreasing from 3.6 mm to 2.3 mm over 5.7 years [14]. Bertoldi (2019) found significant short-term reductions from 2.57 ± 0.61 mm to 2.21 ± 0.68 mm at three months [15]. Ismail (2023) reported stable PD values across all materials (range 2.24-2.75 mm) over three years [18]. El-Ma’aita (2024) observed PD of 2.8 mm at DME sites vs 2.6 mm at controls (p = 0.34) [20]. Farouk (2023) found significantly lower PD for DME than crown-lengthened sites at nine and 12 months (p < 0.05) [19]. Across all studies, PD remained within healthy physiologic limits (1.9-2.8 mm).

Clinical attachment level: CAL was evaluated in three studies [17,19,20]. Muscholl (2022) reported a mean CAL increase of 0.421 mm in DME teeth compared with controls (p = 0.027) [17]. Farouk (2023) observed significantly lower CAL loss in DME compared with crown-lengthening sites at three to 12 months (p < 0.05) [19]. El-Ma’aita (2024) found no significant CAL differences between DME and non-DME restorations [20]. These results suggest that DME does not cause progressive attachment loss and may help preserve periodontal support relative to surgical margin relocation.

Quantitative Pooling Feasibility

Although a quantitative synthesis was planned, meta-analysis was not feasible because the included studies reported outcomes using different scales and statistical units. For example, PD was expressed variably as mean ± SD, categorical improvement, or percentage change, while restoration survival was reported as cumulative proportions over widely differing follow-up periods. Moreover, standard deviations or confidence intervals were missing from several datasets. Consequently, the results are presented as a structured narrative synthesis organized by outcome category (restorative survival, marginal adaptation, and periodontal parameters).

Risk of Bias Among the Included Studies

**Figure 2:**
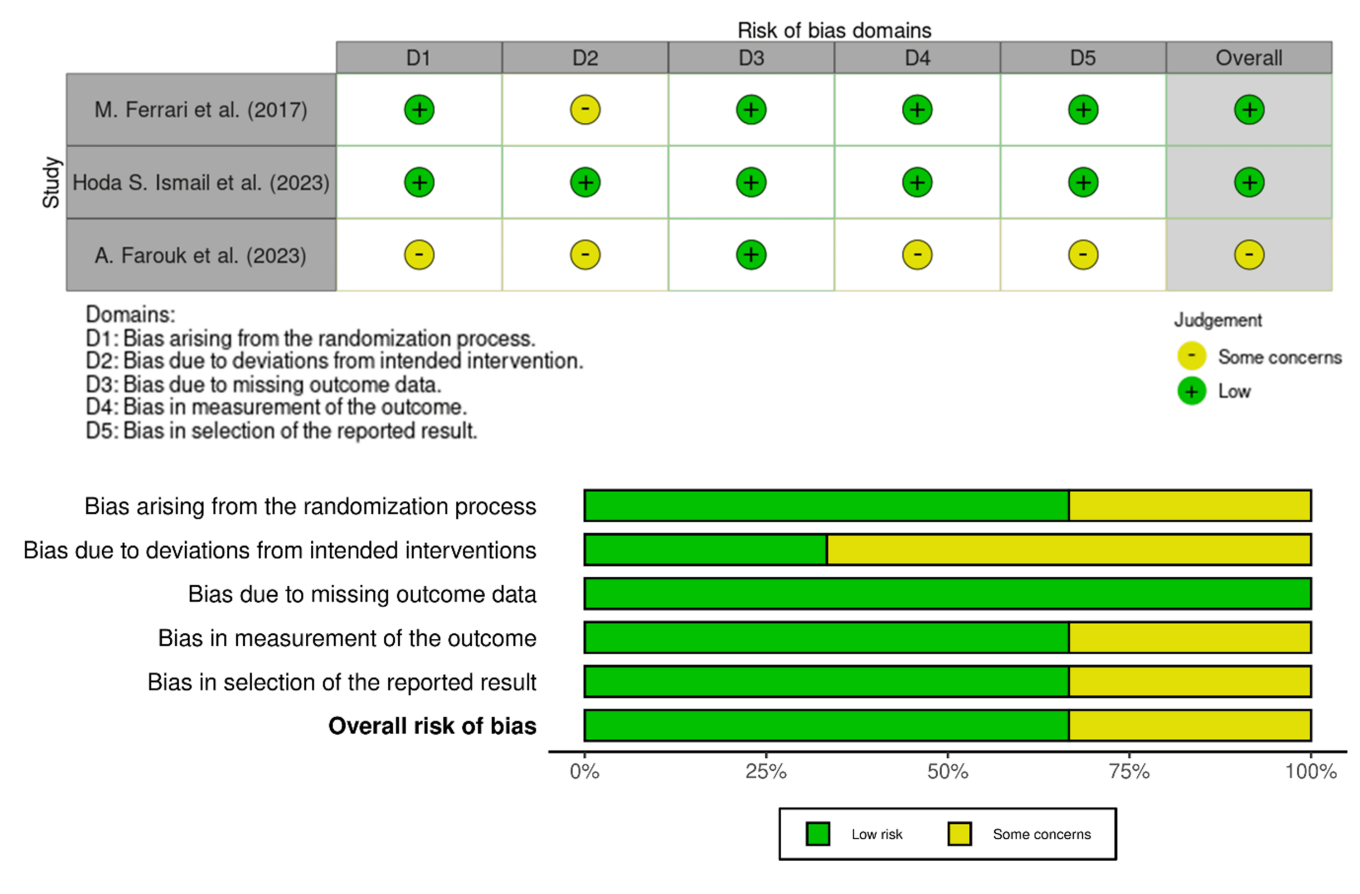
Risk of bias across randomized controlled trials using the Cochrane RoB-2 tool [13,18,19]

The three randomized controlled trials demonstrated overall low to moderate risk of bias when evaluated using the Cochrane RoB-2 tool (Figure 2). Ferrari (2017) was judged to be at low risk across most domains, with only minor concerns regarding participant blinding [13]. Farouk (2023) raised concerns about incomplete details of the randomization process, blinding, and the reporting of numerical data, but no significant threats to validity were identified [19]. Collectively, the RCT evidence is reasonably robust, though the lack of explicit blinding in some studies introduces a small risk of performance or detection bias.

**Figure 3:**
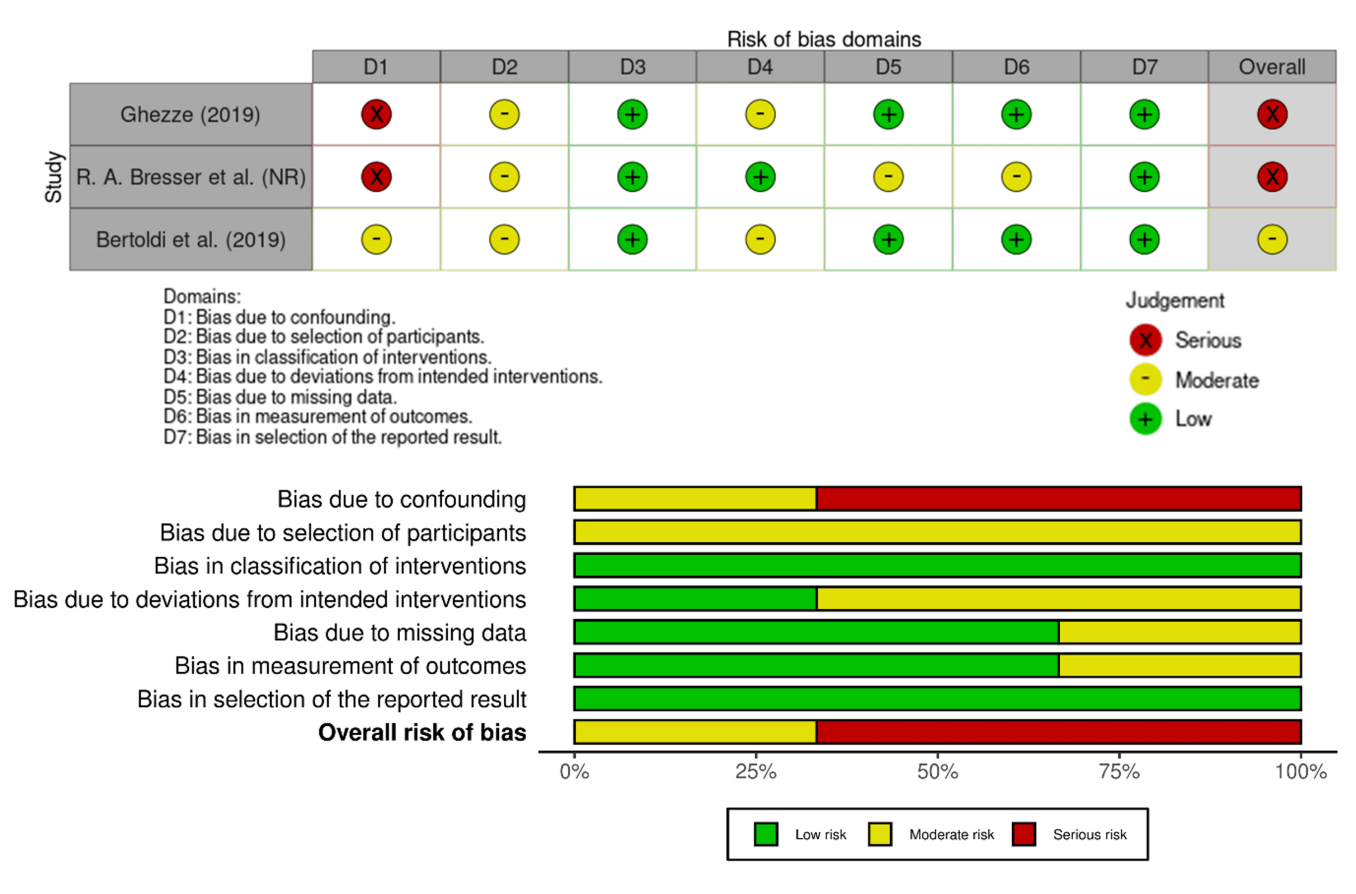
Risk of bias across non-randomized clinical trials using ROBINS-I tool [14-16]

Among the three non-randomized clinical trials, the risk of bias was variable and generally higher (Figure 3). Using ROBINS-I, Ghezze (2019) and Bresser (2019) were rated at serious risk primarily because of potential confounding from uncontrolled patient- or operator-related factors and limitations in participant selection [14,15]. Bertoldi (2019) achieved a moderate risk rating with better control of confounding and standardized outcome assessment, though residual selection bias remained [16]. These findings indicate that results from the non-randomized trials should be interpreted with caution, particularly when comparing periodontal outcomes across different materials or techniques.

The two retrospective observational studies demonstrated low to moderate risk of bias on the Newcastle-Ottawa Scale (Table 2). Muscholl (2022) achieved a high-quality score (7/9), reflecting adequate selection criteria, follow-up, and objective outcome measurement. El-Ma’aita (2024) scored slightly lower (6/9) due to limited control for confounders and a smaller sample size, but still provided credible long-term data [17,20]. Overall, while the retrospective evidence supports the favorable periodontal response to deep margin elevation, the inherent limitations of non-prospective designs necessitate careful integration of these findings with the stronger RCT evidence.

**Table 2:** Risk of bias assessment of the Retrospective Observational Studies using Newcastle–Ottawa Scale (NOS)

Study (Year)	Selection (max 4)	Comparability (max 2)	Outcome (max 3)	Total (max 9)	NOS Quality Summary
Muscholl et al. (2022) [17]	3	1	3	7/9	High quality - Low risk of bias
A. El-Ma’aita et al. (2024) [20]	2	1	3	6/9	Moderate quality -Moderate risk of bias

The domain-specific risk of bias ratings with reasons for downgrades for all the included studies are summaratively listed in Appendix 4.

Certainty of Evidence Assessment

Using the Grading of Recommendations Assessment, Development, and Evaluation (GRADE) framework (Table 3), the overall certainty of evidence for the primary restorative and periodontal outcomes of DME ranged from low to moderate [21]. Evidence for long-term restoration survival was graded as moderate certainty, supported by consistent findings of high survival rates across multiple studies, including one large non-randomized trial with 12-year follow-up, but downgraded for risk of bias and heterogeneity in materials and protocols. PD outcomes were also rated as moderate certainty, as all studies consistently reported healthy PD ranges without progressive attachment loss, though some risk of bias and indirectness were noted. The evidence for BoP and GI was graded as low certainty because of small sample sizes, variability in measurement methods, and early increases in bleeding indices that were not consistently quantified over time. PI showed a similar low-certainty rating due to heterogeneity in restorative materials and operator techniques, as well as imprecision in reporting. Data for CAL were limited to a few studies and were rated as of low certainty, primarily due to serious risk of bias and imprecision. Overall, while the qualitative analysis of the evidence supports DME as a safe and effective approach for managing subgingival margins, the certainty remains constrained by methodological limitations, small sample sizes, and lack of standardized outcome reporting, emphasizing the need for larger, well-designed randomized controlled trials to strengthen future recommendations.

**Table 3:** The certainty of evidence according to the Grading of Recommendations Assessment, Development, and Evaluation (GRADE) framework PD – probing depth, RCT – randomized controlled trial

Outcome Domain	No. of Studies (Design)	Overall Effect/Direction	Key Limitations	GRADE Certainty
Restoration survival/success	5 studies (2 RCTs, 3 non-RCTs, follow-up up to 12 yrs)	Survival consistently high (94–100% at 3–12 yrs) across materials and margin locations	Non-RCT designs with serious risk of bias; small RCT sample sizes; some heterogeneity in materials	Moderate – downgraded for risk of bias and inconsistency
Probing depth	9 studies (3 RCTs, 3 non-RCTs, 2 retrospectives, 1 controlled clinical)	PD remained within healthy range (≈1.9–2.8 mm) with some short-term reductions	Risk of bias in non-RCTs; mild heterogeneity in measurement sites and follow-up	Moderate – downgraded for risk of bias
Bleeding on probing	8 studies (3 RCTs, 3 non-RCTs, 2 retrospectives)	Early transient increase (up to 53%) followed by stabilization or reduction	Serious risk of bias in non-RCTs; moderate inconsistency in magnitude of change	Low – downgraded for risk of bias and inconsistency
Gingival index	5 studies (2 RCTs, 2 non-RCTs, 1 retrospective)	Mild, non-progressive increases over time	Limited RCTs; moderate heterogeneity; small sample sizes	Low – downgraded for risk of bias and imprecision
Plaque index	6 studies (2 RCTs, 3 non-RCTs, 1 retrospective)	Mild early increase with long-term stabilization or decline	Risk of bias and small sample sizes; varied assessment methods	Low – downgraded for risk of bias and imprecision
Clinical attachment level	3 studies (1 RCT, 1 non-RCT, 1 retrospective)	Generally stable	Very small evidence base; serious risk of bias and imprecision	Very Low – downgraded for risk of bias, imprecision, and limited data

Discussion

The present systematic review synthesized the available clinical evidence on DME and its impact on both restorative and periodontal outcomes. Collectively, the findings indicate that DME serves as a viable, conservative alternative to more invasive methods such as surgical crown lengthening or orthodontic extrusion for managing subgingival margins without compromising long-term clinical success [13-20]. The procedure allows for coronal relocation of deep margins, enhancing access for bonding and finishing while maintaining biologic width integrity. It should be noted that no meta-analysis was conducted due to methodological and statistical heterogeneity among studies. Therefore, all findings represent qualitative synthesis rather than pooled effect estimates.

Across the reviewed literature, restorative outcomes were consistently favorable. Survival rates of restorations exceeded 94% in long-term follow-ups extending up to 12 years [15]. Both composite and ceramic restorations demonstrated predictable longevity, although ceramics showed slightly superior performance. The success of DME has been attributed to its adhesive approach, which permits supra- or equigingival margin placement, facilitating improved isolation, bonding, and finishing [4]. The absence of secondary caries reported across studies highlights the ability of DME to create sealed restorative interfaces resistant to bacterial leakage. These findings are consistent with modern adhesive dentistry principles emphasizing minimal intervention and preservation of sound tooth structure. Moreover, even restorations placed on elevated margins exhibited long-term stability, suggesting that meticulous technique and proper material selection can yield durable outcomes regardless of restorative type [22].

Clinically, DME provides several advantages over surgical interventions. It avoids unnecessary removal of periodontal support, reduces chair time, and eliminates the need for multidisciplinary coordination. By enabling easier access for restorative procedures, it enhances marginal adaptation and esthetics. The approach aligns with the principles of minimally invasive restorative dentistry by conserving tooth structure and utilizing adhesive systems with high mechanical and optical performance [23]. However, it remains a technique-sensitive procedure requiring optimal isolation and incremental placement. Any compromise in field control or polymerization may lead to marginal defects and premature failure, emphasizing the importance of adherence to standard protocols and operator skill.

With regard to periodontal parameters, most studies reported stable outcomes following DME. Mean probing depths remained within physiologic limits (1.9-2.8 mm) and showed no evidence of progressive attachment loss [16,19]. In some investigations, slight reductions in probing depths were observed over time, suggesting favorable soft-tissue adaptation when biologic width is respected. Furthermore, clinical attachment levels were found to be superior in DME-treated teeth compared to those treated with surgical crown lengthening after 12 months, indicating that maintaining the original attachment apparatus promotes long-term periodontal stability [19]. Minor transient increases in bleeding on probing and plaque indices were occasionally reported during early follow-ups, likely reflecting temporary inflammatory reactions to newly placed margins [13]. However, these values generally decreased or stabilized at later stages, particularly in patients maintaining effective oral hygiene [14,18]. Proper contouring, polishing, and maintenance of smooth restorative surfaces were emphasized as key factors in minimizing plaque accumulation and gingival inflammation. Additionally, the use of interdental cleaning aids and reinforcement of oral hygiene instructions proved critical in maintaining gingival health around relocated margins [17,24].

Limitations and future scope

Despite promising findings, interpretation of results must consider certain limitations. The included studies displayed heterogeneity in design, margin location, restorative materials, and follow-up durations (ranging from three months to 12 years) [13-20]. Randomization and blinding were inconsistently reported, and several retrospective studies carried inherent risks of bias. Participant demographics and confounding factors such as systemic health or occlusal loading were not uniformly detailed, limiting generalizability. Most outcomes focused on clinical parameters like plaque or probing scores, whereas patient-centered variables such as comfort, esthetic satisfaction, or cost-effectiveness were seldom explored. Future research should therefore prioritize well-designed multicenter randomized controlled trials with standardized DME protocols and long-term follow-ups. Comparative assessments of different restorative materials, including nanohybrids, bioactive composites, and resin-modified glass ionomers, as well as direct versus indirect DME techniques would provide deeper insight. Integration of digital workflows, CAD/CAM-based restorations, and bioactive materials into DME protocols could further refine precision, broaden clinical applicability, and strengthen evidence for its long-term success. Future studies should include quantitative radiographic or CBCT-based assessments of alveolar and crestal bone levels over time to determine whether deep margin elevation influences osseous architecture, since violation of the biologic width has traditionally been associated with bone remodeling and changes in periodontal architecture.

## Conclusions

Within the limitations of the available evidence, the findings of this systematic review suggest that DME can be a promising and conservative option for managing subgingival margins in posterior teeth. The current evidence indicates favorable long-term restoration survival and generally stable periodontal parameters, with only mild, reversible soft-tissue changes when the technique is executed with proper isolation and finishing. Overall, DME appears to offer a biologically compatible and minimally invasive alternative for restoring deep cervical lesions. However, further high-quality clinical studies are needed to confirm these outcomes and refine clinical guidelines.

## Appendices

Appendix 1

**Table 4:** Full Electronic Search Strategies and Search Parameters

Database / Platform	Exact Search String / Boolean Query	Date Executed	Language / Limits	Records Retrieved (n)
PubMed (NCBI)	((“deep margin elevation”[Title/Abstract]) OR (“cervical margin relocation”[Title/Abstract]) OR (“margin relocation”[Title/Abstract])) AND ((“composite resin”[MeSH Terms] OR “composite”[Title/Abstract] OR “resin composite”[Title/Abstract] OR “adhesive”[Title/Abstract]) OR (“resin-modified glass ionomer”[Title/Abstract] OR “RMGI”[Title/Abstract] OR “glass hybrid”[Title/Abstract]) OR (“periodontal”[Title/Abstract] OR “gingival”[Title/Abstract] OR “plaque”[Title/Abstract] OR “probing”[Title/Abstract]) OR (“survival”[Title/Abstract] OR “marginal adaptation”[Title/Abstract])) NOT (“in-vitro”[Title/Abstract] OR “finite element”[Title/Abstract] OR “case report”[Publication Type]) Filters: Humans, English, Dental Journals	15 Aug 2025	English; Humans	38
Scopus (Elsevier)	TITLE-ABS-KEY(“deep margin elevation” OR “cervical margin relocation” OR “margin relocation”) AND TITLE-ABS-KEY(“composite” OR “resin composite” OR “adhesive” OR “resin modified glass ionomer” OR “glass hybrid”) AND TITLE-ABS-KEY(“periodontal” OR “gingival” OR “plaque” OR “probing” OR “survival” OR “adaptation”) AND (LIMIT-TO(LANGUAGE,“English”)) AND (LIMIT-TO(DOCTYPE,“ar”)) AND (LIMIT-TO(SRCTYPE,“j”))	15 Aug 2025	English; Journal Articles	152
Embase (Ovid)	(‘deep margin elevation’/exp OR ‘deep margin elevation’:ti,ab OR ‘cervical margin relocation’:ti,ab OR ‘margin relocation’:ti,ab) AND (‘composite resin’/exp OR composite:ti,ab OR ‘resin composite’:ti,ab OR adhesive:ti,ab OR ‘resin modified glass ionomer’:ti,ab OR ‘glass hybrid’:ti,ab) AND (‘periodontal disease’/exp OR periodontal:ti,ab OR gingival:ti,ab OR plaque:ti,ab OR probing:ti,ab OR survival:ti,ab OR adaptation:ti,ab) NOT (‘in vitro study’/exp OR ‘finite element analysis’/exp OR ‘case report’/exp)	15 Aug 2025	English; Human	152
Web of Science (Core Collection)	TS=(“deep margin elevation” OR “cervical margin relocation” OR “margin relocation”) AND TS=(“composite” OR “resin composite” OR “adhesive” OR “resin modified glass ionomer” OR “glass hybrid”) AND TS=(“periodontal” OR “gingival” OR “plaque” OR “probing” OR “survival” OR “adaptation”) Refined by: LANGUAGE:(English) Indexes: SCI-EXPANDED, SSCI, A&HCI, ESCI Timespan: All years – 2025	15 Aug 2025	English	97
Cochrane CENTRAL	(“deep margin elevation” OR “cervical margin relocation” OR “margin relocation”):ti,ab,kw AND (“composite” OR “adhesive” OR “resin modified glass ionomer” OR “glass hybrid”):ti,ab,kw AND (“periodontal” OR “gingival” OR “plaque” OR “probing” OR “survival” OR “adaptation”):ti,ab,kw Publication Type = Clinical Trial OR RCT	15 Aug 2025	English; Clinical Trials	11
CINAHL (EBSCOhost)	(“deep margin elevation” OR “cervical margin relocation” OR “margin relocation”) AND (“composite” OR “resin composite” OR “adhesive” OR “resin modified glass ionomer” OR “glass hybrid”) AND (“periodontal” OR “gingival” OR “plaque” OR “probing” OR “survival” OR “adaptation”) Limiters: English Language; Human; Peer-Reviewed Journal; Dental Subject Heading	15 Aug 2025	English; Human	33
Google Scholar / Other Grey Sources	“deep margin elevation” OR “cervical margin relocation” “composite” OR “resin modified glass ionomer” OR “adhesive” “periodontal” OR “survival” Filters: English; 2020–2025; Full-text PDF only	15 Aug 2025	English	300
Other Sources (Manual search / Cross-references)	Reference lists of relevant reviews and included studies were screened manually for additional eligible records	15 Aug 2025	English	18

Appendix 2 

**Table 5:** Screening and Eligibility Form

Field	Description / Instructions for Reviewers
Title	Record the full study title as it appears in PubMed or the database.
Abstract	Copy the structured abstract or the first 250 words of the summary.
Authors	List all authors in order as provided.
DOI	Provide the Digital Object Identifier (DOI) number.
DOI Link	Insert the full clickable DOI URL (https://doi.org/…).
Venue / Journal	Record the journal name or source of publication.
Citation Count / Year	Record total citations (if available) and year of publication.
Filename / PDF ID	Enter the filename for the saved full-text PDF.
DME Materials	Yes / No / Maybe – Does the paper explicitly describe using a recognized deep-margin-elevation restorative material (e.g., resin composite, RMGIC, glass hybrid, bulk-fill resin, etc.)?
Reasoning for DME Materials	Justify inclusion or exclusion based on the abstract or methods description.
Study Design	Identify the design: RCT, controlled trial, cohort, case-control, retrospective, or case series (≥ 10 cases).
Reasoning for Study Design	Provide rationale for classification (e.g., “parallel-group RCT comparing DME vs CL”).
Clinical Outcomes Reported	Indicate whether quantifiable clinical outcomes are reported (e.g., PD, CAL, BoP, survival, success rate).
Reasoning for Clinical Outcomes Reported	Explain inclusion based on the presence of measurable outcome variables.
Clinical Study Type	State “Human in vivo,” “In vitro,” or “Animal.” Only Human in vivo is eligible.
Reasoning for Clinical Study Type	Document justification (e.g., “performed in patients receiving DME restorations”).
DME Technique Identification	Yes / No / Maybe – Is the DME or CMR technique explicitly described as a distinct restorative step?
Reasoning for DME Technique Identification	Describe how DME was identified (direct vs indirect technique, margin relocation).
Follow-up Duration	Report follow-up period (months / years). ≥ 6 months required for inclusion.
Reasoning for Follow-up Duration	State evidence from abstract or methods verifying follow-up length.
Human Subjects with DME	Yes / No / Maybe – Confirm involvement of human participants receiving DME restorations.
Reasoning for Human Subjects with DME	Support inclusion with direct evidence from methods.
Sample Size	Enter total cases or number of restorations. ≥ 10 cases required for inclusion.
Reasoning for Sample Size	Note how the number was derived (text / table / methods).
Screening Judgement	Include / Exclude / Maybe – Final decision after first- and second-reviewer consensus.
Reasoning for Screening Judgement	Concise justification summarizing inclusion/exclusion logic.
Screening Score (0–5)	Assign a numeric quality relevance score (0 = poor relevance, 5 = fully meets criteria).

Appendix 3 

**Table 6:** Numeric Summary of Screening and Inclusion

Stage	Description	Number of Records (n)
Identification	Records identified from databases (PubMed = 38; Scopus & Embase = 152; Google Scholar = 300; other sources = 18)	508
	Duplicate records removed before screening	234
	Records removed for other reasons (e.g., incomplete data, inaccessible PDF)	18
Screening	Records screened (titles and abstracts)	256
	Records excluded at title level for clear irrelevance to inclusion criteria	172
	Reports sought for retrieval	84
	Reports not retrieved (abstracts not concerning DME or focused on CAD-CAM prostheses)	66
Eligibility	Full-text reports assessed for eligibility	18
	Reports excluded after full-text review: improper population (n = 7), inadequate/ambiguous data (n = 3)	10
Included	Studies included in the final qualitative synthesis (systematic review)	8
	Studies included in quantitative synthesis (meta-analysis)	Not applicable (data too heterogeneous)

Appendix 4

**Table 7:** Domain-Specific Justifications for Risk-of-Bias Judgments

Study (Year)	Tool Used	Overall Rating / Quality	Domain-wise Ratings and Reasons for Downgrade
M. Ferrari et al. (2017)	Cochrane RoB-2	Low risk of bias	D1: Low – Random sequence and allocation concealment clearly described. D2: Some concerns – Minor deviations from protocol possible but not impactful. D3–D5: Low – Complete data, blinded outcome assessors, and transparent reporting.
Hoda S. Ismail et al. (2023)	Cochrane RoB-2	Low risk of bias	D1–D5: Low – Adequate randomization, adherence to intervention, complete outcome data, reliable measurement, and pre-specified analysis. No downgrades applied.
A. Farouk et al. (2023)	Cochrane RoB-2	Some concerns	D1: Some concerns – Randomization method not fully detailed. D2: Some concerns – Possibility of operator-related deviations. D3–D5: Low – Data completeness and assessor blinding acceptable. Downgraded for unclear allocation and minor procedural variability.
Ghezze (2019)	ROBINS-I	Serious risk of bias	D1: Serious – Confounding not controlled (baseline differences). D2: Moderate – Participant selection not clearly randomized. D3: Low – Intervention classification clear. D4–D7: Moderate–Low – Minor deviations and some incomplete reporting. Overall downgraded for lack of confounder adjustment and unclear inclusion criteria.
R. A. Bresser et al. (NR)	ROBINS-I	Serious risk of bias	D1: Serious – No control for confounding variables. D2: Moderate – Selection bias possible. D3–D7: Low–Moderate – Adequate measurement but unclear blinding. Downgraded due to unadjusted confounding and limited transparency in reporting.
Bertoldi et al. (2019)	ROBINS-I	Moderate risk of bias	D1: Moderate – Minor confounding potential. D2: Moderate – Selection criteria not fully explained. D3–D7: Low – Clear intervention classification, complete data, and low measurement bias. Downgraded slightly for incomplete participant flow description.
Muscholl et al. (2022)	Newcastle–Ottawa Scale	7/9 – High quality / Low risk	Selection: 3/4 – Representative cohort; unclear follow-up source. Comparability: 1/2 – Controlled for age only. Outcome: 3/3 – Standardized assessment and adequate follow-up. Downgraded for limited comparability adjustment.
A. El-Ma’aita et al. (2024)	Newcastle–Ottawa Scale	6/9 – Moderate quality / Moderate risk	Selection: 2/4 – Restricted sample and unclear baseline comparability. Comparability: 1/2 – Single variable adjusted. Outcome: 3/3 – Reliable assessment method. Downgraded for limited representativeness and incomplete confounder control.
